# Impact of the SARS-CoV-2 pandemic on healthy aging and functionality in older Mexican adults: insights from the MHAS cohort

**DOI:** 10.1007/s40520-026-03333-3

**Published:** 2026-02-04

**Authors:** Carmen Arroyo-Quiroz, Silvestre Alavez

**Affiliations:** Departamento de Ciencias de la Salud, Universidad Autónoma Metropolitana, Unidad Lerma, Av. de las Garzas No. 10, Estado de México 52005 Lerma, México

**Keywords:** COVID-19, Healthy aging, Functional decline, Vaccination, Mexico, Older adults

## Abstract

**Background:**

Older adults were among the most affected by the COVID-19 pandemic, facing greater vulnerability to infection, hospitalization, and post-infection sequelae. However, evidence on its multidimensional impact on healthy aging remains limited, particularly in Latin America. This study examined the association of COVID-19 infection, hospitalization, and vaccination with healthy aging and functional impairment among older Mexican adults.

**Methods:**

We analyzed longitudinal data from the Mexican Health and Aging Study (MHAS), comparing pre-pandemic (2018) and post-pandemic (2021) waves. Two outcomes were assessed: the Healthy Aging Score (HAS), a composite indicator of physical, mental, and social functioning (mean = 50, SD = 10), and functional impairment, defined as any limitation in basic or multiple instrumental activities of daily living. Random-effects regression models estimated associations with self-reported COVID-19 infection, hospitalization, and vaccination, adjusting for sociodemographic and health covariates.

**Results:**

The sample included 8,239 participants (mean age = 72.5 years; 55.9% women). Those reporting prior infection were younger and had higher BMI. COVID-19–related hospitalization was significantly associated with lower HAS (β = −1.96; 95% CI − 3.65 to − 0.26). Infection and vaccination were not significantly associated with HAS. However, vaccination was linked to a reduced likelihood of functional impairment (OR = 0.75; 95% CI 0.53 to 0.95).

**Conclusions:**

Hospitalization due to COVID-19 was associated with poorer multidimensional aging outcomes, whereas vaccination appeared protective against functional decline. These findings highlight the importance of preventive strategies and sustained vaccination coverage to preserve functionality and promote healthy aging in post-pandemic populations.

**Graphical Abstract:**

**Figure:**
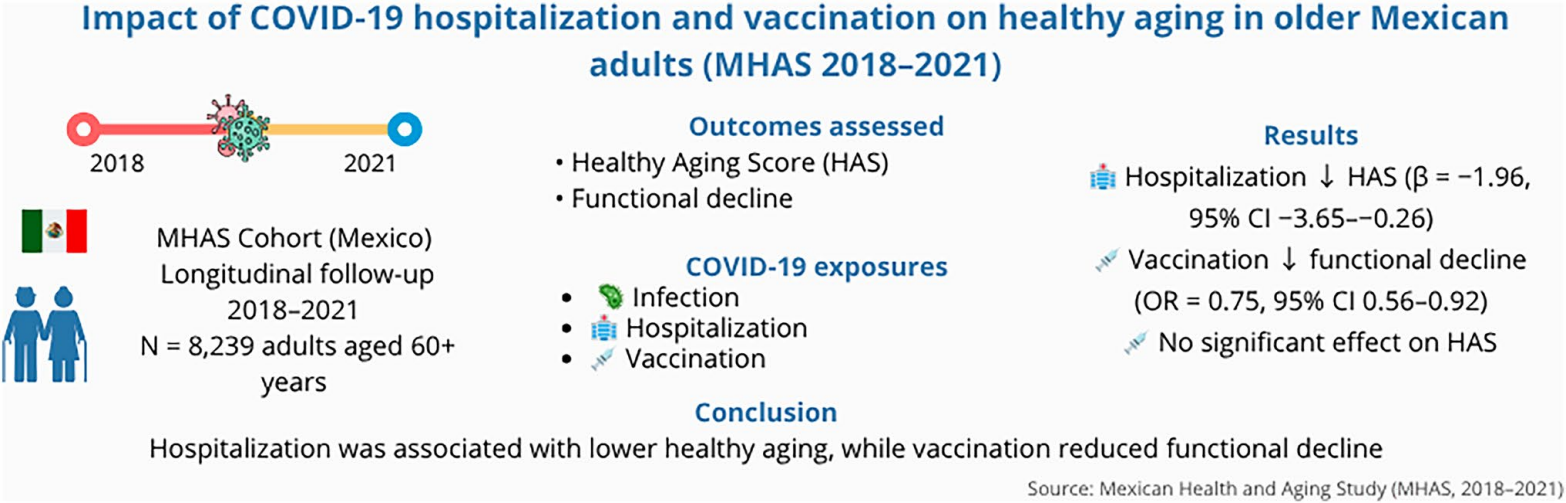
Impact of COVID-19 hospitalization and vaccination on healthy aging among older Mexican adults, MHAS 2018–2021

The graphical abstract summarizes longitudinal findings from 8,239 adults aged 60 years and older in the Mexican Health and Aging Study (2018–2021). COVID-19 hospitalization was associated with lower Healthy Aging Scores, while vaccination was protective against functional decline

**Supplementary Information:**

The online version contains supplementary material available at 10.1007/s40520-026-03333-3.

## Introduction

The COVID-19 pandemic has represented one of the most profound public health challenges of recent decades, with disproportionate consequences for older adults due to age-related physiological vulnerability and comorbid conditions [1, 2]. Beyond the elevated rates of infection, hospitalization, and mortality, accumulating evidence highlights the long-term functional and clinical consequences of SARS-CoV-2 infection in this population [3–5]. Older adults have also shown the highest prevalence of long COVID or persistent post-infection symptoms [6, 7]. These findings suggest that COVID-19 may compromise key domains associated with aging well, such as physical capacity, independence, and overall functioning.

Functional decline and frailty progression have emerged as major post-COVID sequelae among older survivors. In France, nearly half of older patients reported persistent symptoms three months after infection, and one-third were newly classified as frail despite being robust at baseline [8]. Similarly, an Italian cohort reported that over 40% of previously independent older adults either died or lost functional autonomy within six months following hospitalization [9]. A large U.S. study found that nearly one-third of older adults developed at least one new health condition after COVID-19, 11% more than uninfected peers [6]. Consistent with this evidence, hospitalization has been widely used as a proxy for severe COVID-19 among older adults and has been associated with subsequent functional decline, particularly in those aged 70 years and older [10]. These findings point to a broader clinical picture of post-COVID aging characterized by increased frailty, multimorbidity, and functional loss.

In Mexico, the population is aging rapidly, with adults aged 60 years and older now accounting for over 12% of the total population, a proportion that is projected to double within the next three decades [11]. This demographic process is taking place in a context of marked socioeconomic inequality, high levels of informal employment, and a fragmented health system, which influence access to healthcare, social protection, and support services for older adults [12, 13]. These structural conditions are essential to consider when examining health trajectories among older Mexicans.

During the COVID-19 pandemic, these vulnerabilities likely intensified risk in this population. Older adults in Mexico experienced disproportionately high rates of severe disease, hospitalization, and mortality, while simultaneously facing disruptions in routine care, rehabilitation, and community or family support [7, 12, 14]. National vaccination campaigns prioritized older adults and achieved broad coverage, although with regional and socioeconomic heterogeneity [15, 16]. By late 2021, approximately 85–90% of adults aged ≥ 60 years in Mexico had received at least one COVID-19 vaccine dose, and national analyses reported substantial reductions in hospitalizations and deaths following vaccine rollout [16, 17]. Vaccination has been consistently associated with reductions in severe COVID-19 outcomes [18, 19]. However, there is limited evidence on how these pandemic-related experiences relate to broader trajectories of healthy aging in older adults, particularly in Mexico.

Despite emerging evidence on the clinical sequelae of COVID-19, few studies have examined its broader impact on healthy aging using composite indicators that integrate physical, functional, and psychosocial domains, particularly in low- and middle-income countries. According to the World Health Organization, healthy aging refers to “the process of developing and maintaining the functional ability that enables well-being in older age” [20]. Recent international evidence suggests that fewer than one in four older adults achieve healthy aging when assessed comprehensively, highlighting the relevance of monitoring this outcome [21]. Given that COVID-19 and related disruptions may compromise these domains, it is essential to evaluate how pandemic experiences relate to broader aging trajectories. Therefore, this study aimed to analyze the impact of the COVID-19 pandemic on healthy aging and functional status among Mexican older adults and to explore the role of COVID-19 vaccination and hospitalization as relevant health-related variables of interest.

## Materials and methods

### Data sources

Data were drawn from the Mexican Health and Aging Study (MHAS), a nationally representative cohort of adults aged 50 years and older in Mexico [22]. The methodology has been described elsewhere [22, 23]. Data and documentation are publicly available at [www.MHASweb.org].

**Fig. 1:**
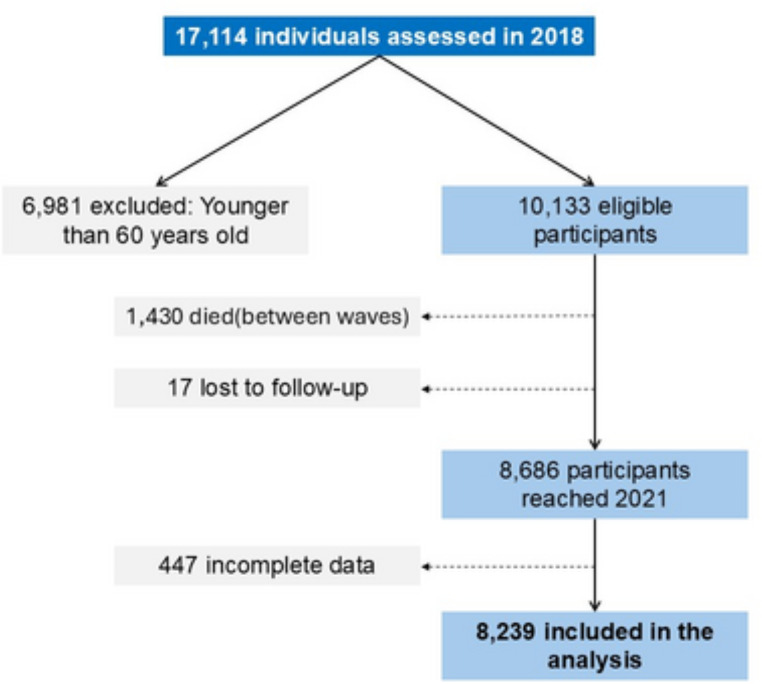
Participant flow diagram showing sample inclusion and follow-up between the 2018 and 2021 waves of the Mexican Health and Aging Study (MHAS)

For this analysis, we included participants interviewed in both the 2018 and 2021 waves. Of 17,114 respondents in 2018, we excluded those younger than 60 years (*n* = 6,981). Among 10,133 eligible participants, 1,430 died and 17 were lost to follow-up, leaving 8,686 survivors in 2021. After excluding incomplete cases, the final analytic sample comprised 8,239 individuals (Fig. 1).

### Study outcomes

Two outcomes were examined: the Healthy Aging Score (HAS) and physical functional impairment assessed through ADL and IADL limitations. The Healthy Aging Score (HAS) was constructed following the methodology by Sánchez-Niubó et al. [24], based on 41 items. The score includes indicators of physical capacity, psychological well-being, sensory function, and social participation. All items were dichotomized to indicate the presence or absence of difficulties, and a two-parameter logistic item response theory (IRT) model was applied. Expected-a-posteriori estimates were transformed into T-scores (mean = 50, SD = 10), representing overall healthy aging, with higher values indicating better healthy aging. In the present study, 39 of the 41 ATHLOS items were operationally available in MHAS and were used to construct the HAS; only two items had no equivalent measure and were therefore not included. Although some individual performance items related to daily functioning are included as indicators of physical capacity, the HAS does not operationalize clinical disability. Functional impairment based on ADL/IADL limitations was analyzed separately as an independent outcome, and chronic diseases were not included in the HAS. A complete list of included items, correspondence with MHAS variables, coding rules, and IRT details is provided in Supplementary Material 1.

Functional status was assessed using basic and instrumental activities of daily living (ADL and IADL). Normal physical function was defined as no limitation in ADL and at most one limitation in IADL, as validated for the Mexican population [25]. ADLs included bathing, dressing, eating, getting into or out of bed, using the toilet, and walking inside the home. IADLs included preparing a hot meal, shopping, taking medications, and managing money. Individuals who did not meet the criteria for normal physical function were classified as having physical functional impairment.

Although a number of individual performance items related to daily functioning are included within the HAS as indicators of physical capacity, the HAS captures a broader multidimensional construct of healthy aging and does not operationalize disability. Therefore, functional impairment was examined separately using an ADL/IADL-based definition, which has been validated and extensively used in studies with Mexican older adults. This distinction is consistent with international conceptual models distinguishing functional capacity from functional disability and ensures comparability with previous literature.

### Main exposure

Self-reported history of COVID-19 infection was assessed in 2021 by the question: *“Since March 2020*,* has a doctor or medical personnel told you that you had or currently have COVID-19?”* Participants additionally reported any COVID-19–related hospitalization and vaccination status (≥ 1 dose). Hospitalization was considered a proxy for severe COVID-19, and vaccination a relevant COVID-related health exposure; both variables are conceptually and epidemiologically justified in the Introduction.

### Covariates

Covariates included sex, age (five-year groups), education, physical activity (≥ 3 times/week), BMI, employment status, and chronic conditions (hypertension, diabetes, heart disease, stroke, obesity, and depression). BMI was initially categorized as < 20 kg/m² (underweight), 20–24.9 kg/m² (normal weight), 25–29.9 kg/m² (overweight), and ≥ 30 kg/m² (obesity), and these categories are presented in Table 1 for descriptive purposes. However, in multivariable models the overweight category was not significantly associated with the outcomes and did not improve model fit; therefore, overweight was combined with the reference group and only obesity (≥ 30 kg/m²) was retained as a separate category in Table 2. All covariates were measured in 2018 and 2021.

**Table 1:** Baseline characteristics of 8,239 Mexican older adults (MHAS 2021) by self-reported COVID-19 infection status

	Total	COVID	Non-COVID	*p*-value*
	*N* = 8239	*n* = 943	*N* = 7296	
Age (years), mean (SD)	72.5 (7.6)	71.1 (6.9)	72.7 (8.8)	< 0.01´
Sex, n(%)				
Male	4434 (44)	390 (41.2)	4044 (44.3)	0.1
Female	5659 (55.9)	551 (58.4)	5108 (55.6)	
Weight status, n(%)				
Underweight	172(2)	7(0.8)	165(2.1)	< 0.01
Normal weight	2915(33.3)	196(23.1)	2719(34.4)	
Overweight	3555(40.6)	377(44.4)	3178(40.2)	
Obesity	1950(22.3)	250(29.4)	1700(21.5)	
Hypertension, n(%)	5190 (51.3)	497 (52.7)	4693 (51.1)	0.38
Diabetes, n(%)	2807 (27.7)	264 (28)	2543 (27.7)	0.86
Cardiac problems, n(%)	901 (8.9)	95 (10.1)	806 (8.8)	0.15
Depressive symptoms, n(%)	2816 (31)	260 (29.4)	2556 (31.2)	0.25
Physical activity, n(%)º	2649 (29.1)	277 (31.2)	2372 (28.8)	0.13
Employment, n(%)	2791 (27.5)	295 (31.3)	2496 (27.2)	0.01
Years of education, mean(SD)	5.3 (4.6)	5.9 (4.8)	5.2 (4.6)	< 0.01´
**Outcomes**				
HAS, mean (SD)	48.6 (10)	49 (9.6)	48.6 (10.1)	0.41
Functionality problems, n(%)	1570 (19.1)	157 (16.6)	1413 (19.4)	0.05

HAS: Healthy Aging Score, *P-value for the comparison COVID-19 survivors and non–COVID-19 survivors, Chi-squared test unless otherwise indicated, ’ Kruskal-Wallis test, ºPhysically active: those who reported to exercise/hard physical work 3 or more times per week

**Table 2:** Estimated associations of COVID-19 status with healthy aging score (HAS) and functional decline.*Regression estimates for the associations between COVID-19 infection*,* hospitalization*,* and vaccination with two outcomes: HAS (continuous) and functional decline (binary).*

Variable	HAS			Functional impairment		
	Coef.	95% CI	*p*-value	OR	95% CI	*p*-value
**Sex (ref: male)**						
Female	-2.09	(-2.45, -1.73)	< 0.001	1.24	(1.08, 1.44)	< 0.001
**Year (ref: 2018)**						
Year 2021	-0.34	(-0.51, -0.17)	< 0.001	1.22	(1.12, 1.34)	< 0.001
**Age group (ref: 60–69 years)**						
70–79	-0.11	(-0.6,0.37)	0.65	0.88	(0.68, 1.15)	0.38
80+	-1.44	(-2.34, -0.54)	< 0.001	1.27	(0.88, 1.63)	0.31
**Health variables (ref: no condition)**						
Hypertension	-1.58	(-1.88, -1.28)	< 0.001	1.34	(1.16, 1.52)	< 0.001
Diabetes	-2.03	(-2.37, -1.69)	< 0.001	1.83	(1.52, 2.11)	< 0.001
Cardiac condition	-2.71	(-3.18, -2.24)	< 0.001	1.96	(1.41, 2.28)	< 0.001
Stroke	-3.13	(-4.03, -2.22)	< 0.001	2.64	(1.93, 3.58)	< 0.001
Obesity	-1.09	(-1.48, -0.71)	< 0.001	1.63	(1.35, 1.95)	< 0.001
Depression	-5.17	(-5.47, -4.86)	< 0.001	3.89	(3.14, 4.72)	< 0.001
Physical activity ≥ 3 times/week	1.46	(1.18, 1.74)	< 0.001	0.61	(0.46, 0.75)	< 0.001
**Socioeconomic variables**						
Employment (ref: not employed)	1.52	(1.20, 1.84)	< 0.001	0.65	(0.48, 0.79)	< 0.001
Income quartile (ref: Q1 – lowest quartile)						
Q2	-0.82	(-1.21, -0.44)	< 0.001	1.22	(1.01, 1.49)	0.04
Q3	-1.07	(-1.52, -0.62)	< 0.001	1.27	(1.03, 1.61)	0.05
Q4	-0.5	(-1.06, 0.07)	0.08	1.06	(0.77, 1.41)	0.71
**COVID VARIABLES (ref: absence of)**						
COVID	-0.25	(-0.78, 0.28)	0.35	0.86	(0.63, 1.02)	0.27
Hospitalization	-1.96	(-3.65, -0.26)	0.02	1.77	(0.88, 2.53)	0.13
Vaccine	0.49	(-0.03, 1.01)	0.07	0.75	(0.56, 0.92)	0.02

HAS = Healthy Aging Score; Q = income quartile; OR = odds ratio. Models adjusted for all covariates listed. The intercept was estimated but is not reported for brevity. Reference categories are indicated in parentheses in the table headers

BMI was initially modeled using four categories. Overweight was not associated with outcomes and was combined with the reference group to improve model parsimony. Only obesity (≥ 30 kg/m²) is shown

Depression was defined using a modified nine-item CES-D scale, with ≥ 5 positive responses indicating depression [26]. All covariates were modeled as time-varying and were updated using information from both 2018 and 2021, consistent with the longitudinal design of the study.

### Statistical analysis

We summarized baseline sociodemographic and lifestyle characteristics as mean ± standard deviation (SD) or median and interquartile range (IQR) for continuous variables, and as percentages for categorical variables. Differences between groups were assessed using chi-square tests for categorical variables and t-tests for continuous variables; the Kruskal–Wallis test was applied when normality assumptions were not met.

Associations between self-reported COVID-19 infection, hospitalization, and vaccination (≥ 1 dose) with healthy aging outcomes were evaluated using random-effects models. For the Healthy Aging Score (HAS), we fitted a random-effects generalized least squares (GLS) model with Mundlak correction, including the individual means of time-varying covariates (mean age, mean income quartile, and mean education level) to separate within- and between-individual variability. For functional impairment, we applied a random-effects logistic regression model with the same specification. Both models adjusted for sex, age group, education, BMI category, physical activity, and chronic conditions (depression, hypertension, diabetes, cardiac disease, obesity, and stroke). Robust standard errors clustered at the individual level were used to account for within-person correlation across observations.

Sensitivity analyses were conducted to evaluate model robustness. First, we re-estimated models excluding major chronic diseases (depression, hypertension, and diabetes). Second, we restricted the sample to participants younger than 80 years. Third, outliers in HAS, functional outcomes, and BMI were excluded. Fourth, we incorporated interaction terms between vaccination and sex or age. Finally, fixed-effects models were fitted for comparison with random-effects estimates.

All analyses were conducted using Stata version 18.5 (StataCorp, College Station, TX).

### Ethics approval and consent to participate

This study is a secondary analysis of de-identified data from the Mexican Health and Aging Study (MHAS). The MHAS protocol received ethical approval from the Institutional Review Board of the University of Texas Medical Branch in the United States and the Ethics Committee of the National Institute of Public Health in Mexico. All participants provided informed consent prior to data collection in each wave.

## Results

Table 1 presents the main characteristics of the study sample, which comprised 8,239 participants, 55.9% of whom were women. At baseline, the mean age was 72.5 years (SD = 7.6). Among participants, 62.9% were classified as overweight or obese, 27.7% had diabetes, 51.3% had hypertension, and 8.9% reported cardiac disease. Regarding socioeconomic variables, 27.5% were employed, and the mean years of education were 5.3 (SD = 4.6).

Most variables showed significant differences between participants with and without a history of COVID-19 infection. Compared with non-COVID-19 participants, those who reported previous infection were younger, had higher BMI, and more frequently reported employment and higher education. In 2018, the COVID-19 group also had fewer functional impairments (16.6% vs. 19.4%, *p* = 0.05) and slightly higher HAS, although this difference was not statistically significant (49.0 vs. 48.6, *p* = 0.41).

**Fig. 2:**
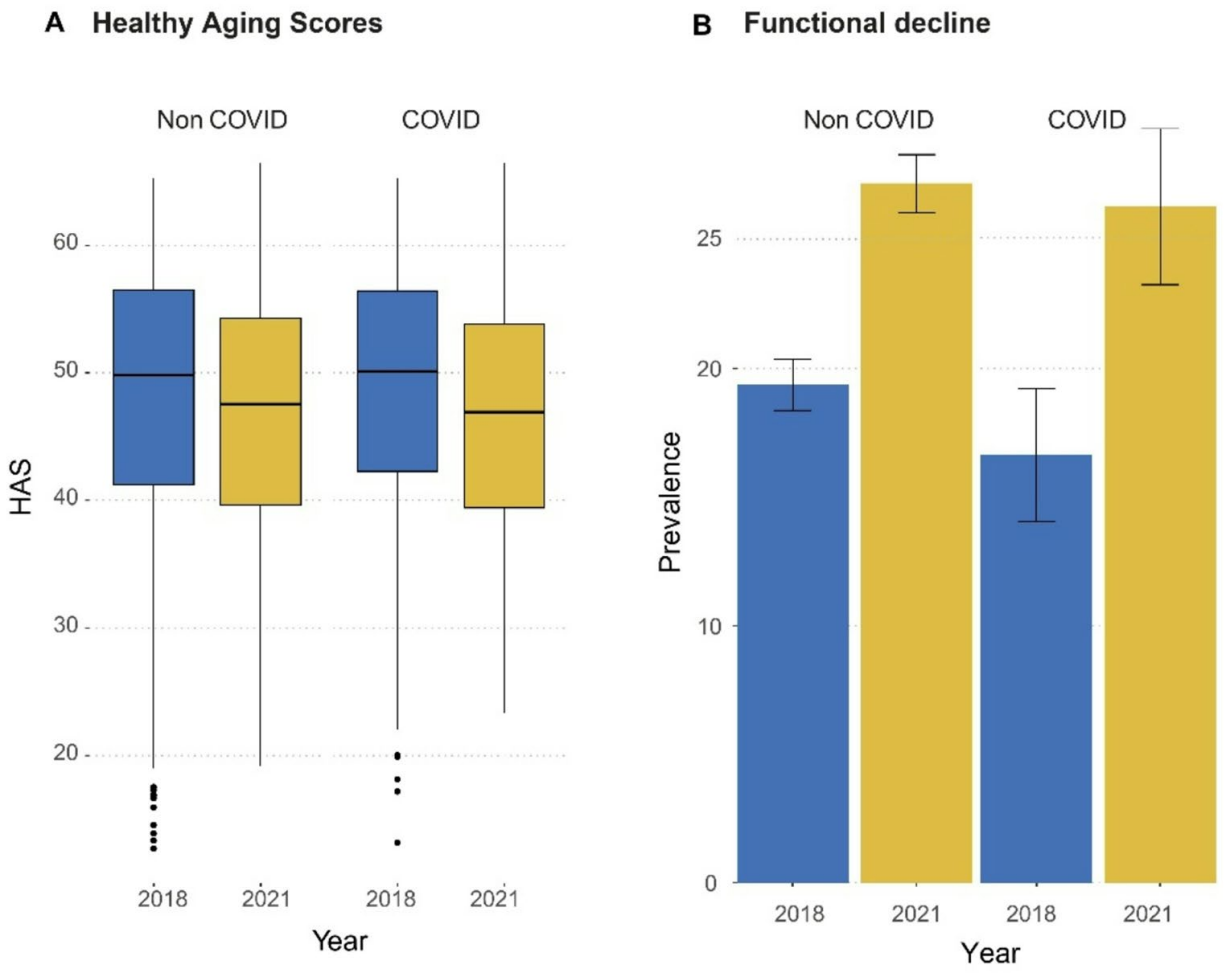
Healthy Aging Score (HAS) and prevalence of functional decline among older Mexican adults in 2018 and 2021. Panel A displays mean HAS scores by COVID-19 infection status across both waves. Panel B shows the proportion of participants with functional decline, highlighting increased prevalence in both groups by 2021. Error bars represent standard deviations

HAS T-scores were computed for all participants with two available measurements (Fig. 2A). The mean HAS was 48.6 (SD = 10.0) in 2018 and 47.0 (SD = 10.0) in 2021, representing an average decrease of − 1.62 points (SD = 8.7), equivalent to a − 1.4% change over the study period.

When comparing groups, individuals with prior COVID-19 infection showed higher HAS at baseline (48.9 vs. 48.6, *p* < 0.001) but not at follow-up (46.6 vs. 47.0, *p* = 0.17). The mean percentage change was slightly greater among the COVID-19 group (− 4.8%) than in the non-COVID group (− 3.1%), although the difference was not statistically significant (*p* = 0.19).

Regarding functional impairment (Fig. 2B), its prevalence increased from 19.1% in 2018 to 27.1% in 2021 (*p* < 0.001). Among non-COVID participants, impairment rose from 19.4% to 27.2% (≈ 40% increase), while in the COVID-19 group it increased from 16.7% to 26.3% (≈ 58% increase). Both within-group changes were statistically significant (*p* < 0.001).

The results of multivariate models for HAS and functional impairment, adjusted for sex, age, chronic conditions, physical activity, employment, and obesity, are presented in Table 2.

For HAS, protective effects were observed for regular exercise (β = 1.46, 95% CI: 1.18–1.74) and employment (β = 1.52, 95% CI: 1.20–1.84). Negative associations were found for female sex (β = −2.09, 95% CI: −2.45, − 1.73), older age groups, hypertension (β = −1.58, 95% CI: −1.88, − 1.28), diabetes (β = −2.03, 95% CI: −2.37, − 1.69), cardiac disease (β = −2.71, 95% CI: −3.18, − 2.24), obesity (β = −1.09, 95% CI: −1.48, − 0.71), and depression (β = −5.17, 95% CI: −5.47, − 4.86).

COVID-19 infection and vaccination were not significantly associated with HAS, whereas hospitalization due to COVID-19 was negatively associated (β = −1.96, 95% CI: −3.65, − 0.26). Between-individual effects were significant for mean age (β = −2.82, 95% CI: −3.33, − 2.31) and mean income quartile (β = 1.09, 95% CI: 0.83, 1.36), suggesting that higher long-term income is associated with better healthy aging outcomes.

For functional impairment, the direction and significance of most covariates were consistent with those observed for HAS, although some differences in magnitude were noted. Depression showed the strongest association (OR = 3.88, 95% CI: 3.14–4.72, *p* < 0.001), followed by stroke (OR = 2.64, 95% CI: 1.93–3.58, *p* < 0.001) and diabetes (OR = 1.83, 95% CI: 1.52–2.11, *p* < 0.001). Female sex, older age, and chronic conditions (hypertension, cardiac disease, obesity, stroke) were significantly associated with increased odds of functional impairment. Conversely, regular exercise (OR = 0.60, 95% CI: 0.46–0.75, *p* < 0.001) and employment (OR = 0.52, 95% CI: 0.39–0.65, *p* < 0.001) were protective. Neither self-reported COVID-19 infection nor hospitalization was significantly associated with functional impairment, whereas vaccination showed a protective effect (OR = 0.75, 95% CI: 0.53–0.95, *p* = 0.02).

### Sensitivity Analyses

Sensitivity analyses confirmed the robustness of the results (Supplementary Material 1–2). Excluding depression, hypertension, diabetes, or participants aged ≥ 80 years resulted in only minor changes in effect sizes without altering direction or significance. Fixed-effects models, particularly for functional impairment, retained fewer cases and slightly affected some estimates. Removing extreme values of HAS, functional outcomes, and BMI confirmed that results were not driven by outliers. Interaction terms for vaccination with sex and age were non-significant. Despite the omission of variables with no within-group variation (e.g., sex, age group, income quartile), associations with chronic conditions, physical activity, and employment status remained consistent. Overall, findings across datasets supported the stability of the original models.

## Discussion

In this nationally representative study of older Mexican adults, we examined the association of COVID-19 infection, hospitalization, and vaccination with healthy aging and functional status. We found that COVID-19–related hospitalization, as an indicator of COVID-19 severity, was significantly associated with lower Healthy Aging Scores (HAS), while vaccination was protective against functional impairment. These findings suggest that severe COVID-19 may accelerate declines in multidimensional health, whereas vaccination contributes to preserving autonomy and functionality in later life. These patterns are particularly relevant in Mexico, where vaccination coverage among adults aged ≥ 60 years reached over 80% during 2021, yet regional and socioeconomic heterogeneity persists, implying that not all older adults benefited equally from immunization efforts [15, 17].

The Healthy Aging Score (HAS) captures a broad construct of health, integrating physical, cognitive, and psychosocial domains. A lower HAS indicates reduced physiological reserve and resilience—factors closely linked to frailty, disability, and mortality risk. The observed negative association between COVID-19 hospitalization and HAS therefore signals potential long-term vulnerability in older adults recovering from severe infection. These results align with clinical observations of post-COVID functional loss and with the conceptual overlap between post-acute sequelae and frailty syndromes [27]. In line with recent evidence showing that older adults hospitalized with COVID-19 experience greater functional loss and poorer prognosis than younger patients, but can still benefit substantially from inpatient rehabilitation [10], our findings suggest that preserving functional reserve after severe infection is a key component of healthy aging trajectories.

The Healthy Aging Score (HAS) is consistent with the WHO definition of healthy aging as the maintenance of functional ability determined by intrinsic capacity and environmental factors [28]. Evidence from ATHLOS and related studies shows that multidimensional indicators of this type are comparable across populations and predict mortality and functional outcomes [24, 29]. Similar approaches applied in the Mexican Health and Aging Study have also identified heterogeneous healthy aging trajectories, reinforcing the relevance of multidimensional indicators in this context [30]. Thus, the HAS is suitable for interpreting the potential long-term impact of COVID-19 on aging trajectories.

Our findings complement previous research documenting persistent symptoms and reduced functional capacity among older survivors of COVID-19 [8, 9, 31]. Studies in Europe and North America have reported that up to 40–50% of older adults hospitalized for COVID-19 experience new or worsened limitations in daily functioning, mobility, or cognitive performance within months of discharge [9, 32]. The mechanisms underlying these changes are multifactorial and may include prolonged inflammation, endothelial dysfunction, deconditioning, and exacerbation of pre-existing comorbidities [33, 34]. Such biological and behavioral pathways can compromise the capacity to recover homeostasis after stressors, accelerating transitions from robustness to vulnerability—one of the key determinants of healthy aging trajectories.

Although COVID-19–related exposures were the primary focus of this study, several covariates consistently emerged as robust predictors of healthy aging and functional outcomes. Female sex, depression, diabetes, and cardiovascular conditions (including cardiac disease and stroke) were all significantly associated with lower HAS scores and increased functional impairment. These findings align with `gender-health paradox´ whereby women, despite their greater longevity, frequently experience higher disability burden in later life, even after controlling for age and other risk factors [35–37]; that depression substantially contributes to functional decline and reduced resilience [38, 39]; and that cardiometabolic diseases accelerate biological aging, frailty, and disability trajectories [40–42]. Recent analyses using MHAS and other Mexican data have also documented widening inequalities in basic and instrumental activities of daily living by wealth and gender, with women and poorer older adults experiencing higher and increasing levels of functional limitations over time [43]. By acknowledging these underlying factors, we can contextualize our results within the long-standing health disparities that continue to shape the lives of older adults across Mexico.

The protective association of vaccination with functional outcomes underscores the broader benefits of immunization beyond infection prevention. COVID-19 vaccines have been shown to reduce disease severity, hospitalization, and mortality in older adults [44], and accumulating evidence suggests that vaccinated individuals who become infected exhibit milder and shorter post-COVID sequelae [45, 46]. From a geriatric perspective, maintaining vaccination coverage is an essential component of preserving function and delaying frailty progression. In the Mexican context—where vaccination coverage in older adults exceeded 80% by mid-2021 [17]—these results reinforce the role of sustained immunization and booster campaigns as tools to protect multidimensional health in aging populations. In Mexico, although vaccination coverage among older adults was high, it was not evenly distributed. Evidence shows that vaccination uptake was lower among older adults with fewer economic resources, lower education, and more limited access to health services, particularly in rural and disadvantaged contexts [15, 44, 45]. In Mexico City, for example, around 7–8% of older adults did not receive a first vaccine dose despite universal eligibility, mainly due to misinformation, vaccine hesitancy, socioeconomic barriers, and household food insecurity [15]. These inequities are relevant to interpreting our findings: they suggest that the protective association between vaccination and functional outcomes likely reflects not only biomedical protection but also underlying social advantage [47]. Older adults with better socioeconomic conditions may have been more likely to access vaccination and, simultaneously, to maintain health care continuity, rehabilitation, nutrition, and social support during the pandemic [15, 47]. Therefore, the benefits of vaccination may have compounded existing social gradients in aging, reinforcing the need for equity-oriented vaccination policies and targeted support for vulnerable older adults.

Interestingly, self-reported COVID-19 infection was not associated with either HAS or functional impairment. This lack of association likely reflects measurement limitations. Only 10% of participants reported infection, far below the estimated 25% national seroprevalence [17, 48]. Many infections among older adults may have been asymptomatic or unrecognized, as a substantial proportion of SARS-CoV-2 infections occur without symptoms or go undetected in surveillance systems [48, 49]. Moreover, self-reported variables introduce recall bias and differential misclassification, particularly in cognitively impaired individuals. In contrast, hospitalization data are less subject to reporting errors and represent severe cases, explaining the stronger associations observed for this exposure.

The overlap between post-COVID symptoms and aging-related conditions complicates causal attribution. Chronic fatigue, reduced endurance, dyspnea, and cognitive difficulties are common to both long COVID and geriatric syndromes. Recent studies have emphasized that acute SARS-CoV-2 infection can precipitate decompensation in multiple systems, including cardiovascular, metabolic, and neurological pathways [50, 51]. The convergence of these processes may amplify frailty and accelerate the decline of functional reserve, leading to lower HAS scores over time.

Our results also highlight the importance of contextual factors in Latin America. Older adults in Mexico often face high burden of multimorbidity, poverty, and limited access to health care services [12]. Moreover, external shocks such as the COVID-19 pandemic have exacerbated vulnerabilities in nutritional and functional status among older adults [52]. In addition, the Mexican health system is highly fragmented, and access to services remains unequal across socioeconomic and geographic groups [13]. These structural barriers likely influence not only the probability of receiving vaccination and timely COVID-related care, but also access to rehabilitation and follow-up services that are essential to preserving functionality after severe illness. The pandemic further disrupted chronic-disease management and social support, conditions that are strongly linked to functional loss. The HAS, by capturing biopsychosocial dimensions, provides a valuable lens to monitor how such structural inequities translate into differential aging trajectories. Integrating functional and psychosocial indicators into national surveillance could strengthen early identification of individuals at risk of accelerated aging.

More broadly, evidence from MHAS and Mexican national health surveys shows that access to health care, hospitalization, and preventive service use among older adults are strongly patterned by health insurance coverage, socioeconomic status, and urban–rural residence, highlighting ongoing structural inequities within [43, 53, 54]. These factors likely shaped not only COVID-19 exposure and vaccine accessibility but also the capacity for rehabilitation and functional recovery. Consequently, employing the Healthy Aging Scale (HAS) enables an assessment of how structural and social determinants manifest as unequal aging trajectories, extending the analysis beyond purely clinical outcomes.

Some limitations should be considered. First, all measures, including COVID-19 exposure, were self-reported and subject to recall bias. The timing between infection and assessment could not be determined, and effects may have attenuated among early cases. Second, unmeasured confounding (e.g., nutrition, medication use, sleep quality) could influence results. Third, the number of confirmed COVID-19 cases was smaller than national estimates, reducing statistical power for subgroup analyses. Despite these limitations, our study has notable strengths: the use of a nationally representative cohort, pre-pandemic baseline data, and a validated multidimensional indicator of healthy aging. These features enable a more robust evaluation of pandemic-related changes in functionality and resilience among older adults.

Clinically, our findings suggest that post-hospitalization follow-up and functional assessment should be routine for older adults recovering from COVID-19. Rehabilitation programs addressing mobility, endurance, and psychosocial well-being may mitigate the long-term effects of severe infection. Public-health strategies should also emphasize booster vaccination and targeted outreach to socioeconomically disadvantaged older adults, who may experience greater barriers to care and recovery. From a research perspective, future studies should incorporate objective measures of physical performance (e.g., grip strength, gait speed), biomarkers of inflammation, and longer follow-up to clarify the biological mechanisms linking COVID-19 severity to healthy aging outcomes. More broadly, our findings reinforce the relevance of healthy aging as a policy framework, highlighting the need to protect functional capacity, resilience, and autonomy among older adults in the post-pandemic context.

## Conclusions

COVID-19 hospitalization was associated with lower Healthy Aging Scores, indicating multidimensional health deterioration among older adults, while vaccination was protective against functional impairment. These findings reinforce the clinical importance of preventing severe infections and maintaining vaccination coverage to sustain functionality, independence, and healthy aging. As health systems adapt to the post-pandemic era, continuous monitoring of functional and psychosocial domains in older adults will be critical to mitigate the long-term impact of COVID-19 on aging trajectories.

## Supplementary Information

Below is the link to the electronic supplementary material.

Supplementary Material 1

Supplementary Material 2

## Data Availability

No datasets were generated or analysed during the current study.

## References

[1] Kang SJ, Jung SI (2020) Age-Related morbidity and mortality among patients with COVID-19. Infect Chemother 52(2):154–164. 10.3947/ic.2020.52.2.154 32537961 10.3947/ic.2020.52.2.154 PMC7335648

[2] Mehraeen E, Karimi A, Barzegary A, Vahedi F, Afsahi AM, Dadras O et al (2020) Predictors of mortality in patients with COVID-19-a systematic review. Eur J Integr Med 40:101226. 10.1016/j.eujim.2020.101226 33101547 10.1016/j.eujim.2020.101226 PMC7568488

[3] Dadras O, SeyedAlinaghi S, Karimi A, Shamsabadi A, Qaderi K, Ramezani M et al (2022) COVID-19 mortality and its predictors in the elderly: A systematic review. Health Sci Rep 5(3):e657. 10.1002/hsr2.657 35620541 10.1002/hsr2.657 PMC9125886

[4] Daitch V, Yelin D, Awwad M, Guaraldi G, Milic J, Mussini C et al (2022) Characteristics of long COVID among older adults: a cross-sectional study. Int J Infect Dis. 10.1016/j.ijid.2022.09.035 36191820 10.1016/j.ijid.2022.09.035

[5] Promislow DEL (2020) A geroscience perspective on COVID-19 mortality. J Gerontol Biol Sci Med Sci 75(9):e30–e3. 10.1093/gerona/glaa094 10.1093/gerona/glaa094 PMC7184466 32300796

[6] Cohen K, Ren S, Heath K, Dasmarinas MC, Jubilo KG, Guo Y et al (2022) Risk of persistent and new clinical sequelae among adults aged 65 years and older during the post-acute phase of SARS-CoV-2 infection: retrospective cohort study. BMJ 376:e068414. 10.1136/bmj-2021-068414 35140117 10.1136/bmj-2021-068414 PMC8828141

[7] Gonzalez-Gonzalez C, Orozco-Rocha K, DeGraff DS, Samper-Ternent R, Wong R (2023) COVID-19 and mental health outcomes of older adults: evidence from Mexico. Health Aff (Millwood) 42(12):1675–1680. 10.1377/hlthaff.2023.00698 38048498 10.1377/hlthaff.2023.00698

[8] Prampart S, Le Gentil S, Bureau ML, Macchi C, Leroux C, Chapelet G et al (2022) Functional decline, long term symptoms and course of frailty at 3-months follow-up in COVID-19 older survivors, a prospective observational cohort study. BMC Geriatr 22(1):542. 10.1186/s12877-022-03197-y 35768781 10.1186/s12877-022-03197-y PMC9244035

[9] Okoye C, Calsolaro V, Calabrese AM, Zotti S, Fedecostante M, Volpato S et al (2022) Determinants of Cause-Specific mortality and loss of independence in older patients following hospitalization for COVID-19: the GeroCovid outcomes study. J Clin Med 11(19). 10.3390/jcm11195578 10.3390/jcm11195578 PMC9571114 36233447

[10] Lapo HM, Sardeli AV, Mariano LO, Howroyd FJ, Sokoll PR, Sapey E et al (2024) Functionality loss due to COVID-19 hospitalisation in older adults recovers with inpatient rehabilitation: A systematic review and meta-analysis. Exp Gerontol 198:112617. 10.1016/j.exger.2024.112617 39490696 10.1016/j.exger.2024.112617

[11] CONAPO (2012) Proyecciones de La Población 2010–2050. Mexico City. Consejo Nacional de Poblacion (CONAPO), Mexico, pp 78–79

[12] Rivera-Hernandez M, Ferdows NB, Kumar A (2021) The impact of the COVID-19 epidemic on older adults in rural and urban areas in Mexico. J Gerontol B Psychol Sci Soc Sci 76(7):e268–e74. 10.1093/geronb/gbaa227 33367752 10.1093/geronb/gbaa227 PMC7798580

[13] Gomez-Dantes O, Flamand L, Cerecero-Garcia D, Morales-Vazquez M, Servan-Mori E (2023) Origin, impacts, and potential solutions to the fragmentation of the Mexican health system: a consultation with key actors. Health Res Policy Syst 21(1):80. 10.1186/s12961-023-01025-2 37525130 10.1186/s12961-023-01025-2 PMC10388521

[14] Bello-Chavolla OY, Fermin-Martinez CA, Ramirez-Garcia D, Vargas-Vazquez A, Fernandez-Chirino L, Basile-Alvarez MR et al (2024) Prevalence and determinants of post-acute sequelae after SARS-CoV-2 infection (Long COVID) among adults in Mexico during 2022: a retrospective analysis of nationally representative data. Lancet Reg Health Am 30:100688. 10.1016/j.lana.2024.100688 38327277 10.1016/j.lana.2024.100688 PMC10847769

[15] Gaitan-Rossi P, Mendez-Rosenzweig M, Garcia-Alberto E, Vilar-Compte M (2022) Barriers to COVID-19 vaccination among older adults in Mexico City. Int J Equity Health 21(1):85. 10.1186/s12939-022-01685-6 35717236 10.1186/s12939-022-01685-6 PMC9206538

[16] Bello-Chavolla OY, Antonio-Villa NE, Valdes-Ferrer SI, Fermin-Martinez CA, Fernandez-Chirino L, Vargas-Vazquez A et al (2023) Effectiveness of a nationwide COVID-19 vaccination program in Mexico against symptomatic COVID-19, hospitalizations, and death: a retrospective analysis of National surveillance data. Int J Infect Dis 129:188–196. 10.1016/j.ijid.2023.01.040 36775188 10.1016/j.ijid.2023.01.040 PMC9918316

[17] Basto-Abreu A, Carnalla M, Torres-Ibarra L, Sanchez-Pajaro A, Romero-Martinez M, Martinez-Barnetche J et al (2023) SARS-CoV-2 Seroprevalence and vaccine coverage from August to November 2021: A nationally representative survey in Mexico. J Med Virol 95(8):e29038. 10.1002/jmv.29038 37615363 10.1002/jmv.29038

[18] Wong BK, Mabbott NA (2024) Systematic review and meta-analysis of COVID-19 mRNA vaccine effectiveness against hospitalizations in adults. Immunother Adv 4(1):ltae011. 10.1093/immadv/ltae011 39703784 10.1093/immadv/ltae011 PMC11655844

[19] Rojas-Castro M, Verdasca N, Monge S, De Mot L, Trobajo-Sanmartin C, Duffy R et al (2025) COVID-19 vaccine effectiveness against hospitalization in older Adults, VEBIS hospital Network, Europe, September 2024-May 2025. Influenza Other Respir Viruses 19(11):e70191. 10.1111/irv.70191 41290396 10.1111/irv.70191 PMC12646827

[20] World Health O (2020) Decade of healthy ageing 2020–2030. World Health Organization, Geneva

[21] Tang L, Rasudin NSB, Dong Y, Yusuf A (2025) Prevalence and related factors of healthy aging: A systematic review and meta-analysis. Belitung Nurs J 11(5):504–516. 10.33546/bnj.3977 41059004 10.33546/bnj.3977 PMC12498238

[22] Wong R, Michaels-Obregon A, Palloni A, Gutierrez-Robledo LM, Gonzalez-Gonzalez C, Lopez-Ortega M et al (2015) Progression of aging in mexico: the Mexican health and aging study (MHAS) 2012. Salud Publica Mex 57(Suppl 1):S79–89 26172238 10.21149/spm.v57s1.7593 PMC4705907

[23] MHAS MHaAS (2012) Data Files and Documentation (public use): Mexican Health and Aging Study, Database. https://www.MHASweb.org. Retrieved from on 11 Mar 2019

[24] Sanchez-Niubo A, Forero CG, Wu YT, Gine-Vazquez I, Prina M, De La Fuente J et al (2021) Development of a common scale for measuring healthy ageing across the world: results from the ATHLOS consortium. Int J Epidemiol 50(3):880–892. 10.1093/ije/dyaa236 33274372 10.1093/ije/dyaa236 PMC8271194

[25] Mejia-Arango S, Gutierrez LM (2011) Prevalence and incidence rates of dementia and cognitive impairment no dementia in the Mexican population: data from the Mexican health and aging study. J Aging Health 23(7):1050–1074. 10.1177/0898264311421199 21948770 10.1177/0898264311421199 PMC3557523

[26] Aguilar-Navarro G, Á-FA, García-Mayo J (2007) Validez y confiabilidad Del cuestionario Del ENASEM Para La depresión En Adultos mayores. Salud Publica De México 49(4):256–262 17710274 10.1590/s0036-36342007000400005

[27] Mansell V, Hall Dykgraaf S, Kidd M, Goodyear-Smith F (2022) Long COVID and older people. Lancet Healthy Longev 3(12):e849–e54. 10.1016/S2666-7568(22)00245-8 36480981 10.1016/S2666-7568(22)00245-8

[28] World Health O (2015) World report on ageing and health. World Health Organization, Geneva

[29] Beard JR, Jotheeswaran AT, Cesari M, Araujo de Carvalho I (2019) The structure and predictive value of intrinsic capacity in a longitudinal study of ageing. BMJ Open 9(11):e026119. 10.1136/bmjopen-2018-026119 31678933 10.1136/bmjopen-2018-026119 PMC6830681

[30] Daskalopoulou C, Koukounari A, Wu YT, Terrera GM, Caballero FF, de la Fuente J et al (2019) Healthy ageing trajectories and lifestyle behaviour: the Mexican health and aging study. Sci Rep 9(1):11041. 10.1038/s41598-019-47238-w 31363117 10.1038/s41598-019-47238-w PMC6667468

[31] Carfi A, Bernabei R, Landi F, Gemelli Against C-P-ACSG (2020) Persistent symptoms in patients after acute COVID-19. JAMA 324(6):603–605. 10.1001/jama.2020.12603 32644129 10.1001/jama.2020.12603 PMC7349096

[32] Walle-Hansen MM, Ranhoff AH, Mellingsaeter M, Wang-Hansen MS, Myrstad M (2021) Health-related quality of life, functional decline, and long-term mortality in older patients following hospitalisation due to COVID-19. BMC Geriatr 21(1):199. 10.1186/s12877-021-02140-x 33752614 10.1186/s12877-021-02140-x PMC7983098

[33] Xie Y, Al-Aly Z (2022) Risks and burdens of incident diabetes in long COVID: a cohort study. Lancet Diabetes Endocrinol 10(5):311–321. 10.1016/S2213-8587(22)00044-4 35325624 10.1016/S2213-8587(22)00044-4 PMC8937253

[34] Xu E, Xie Y, Al-Aly Z (2023) Risks and burdens of incident dyslipidaemia in long COVID: a cohort study. Lancet Diabetes Endocrinol 11(2):120–128. 10.1016/S2213-8587(22)00355-2 36623520 10.1016/S2213-8587(22)00355-2 PMC9873268

[35] Arroyo-Quiroz C, Brunauer R, Alavez S (2023) Education and employment have a sex-dependent effect on healthy aging in Mexico. Geriatr Gerontol Int 23(12):980–981. 10.1111/ggi.14731 37961039 10.1111/ggi.14731 PMC11503573

[36] Correa L, Gomes CDS, Camara S, Barbosa JFS, Azevedo IG, Vafaei A et al (2023) Gender-Specific associations between Late-Life disability and socioeconomic status: findings from the international mobility and aging study (IMIAS). Int J Environ Res Public Health 20(4). 10.3390/ijerph20042789 10.3390/ijerph20042789 PMC9956095 36833484

[37] Augustsson E, Rehnberg J, Simmons C, Rodrigues R, Kadi S, Ilinca S et al (2023) Can sex differences in old age disabilities be attributed to socioeconomic conditions? Evidence from a mapping review of the literature. J Popul Ageing 16(3):761–780. 10.1007/s12062-022-09395-1

[38] Wang S, Yu M, Huang W, Wang T, Liu K, Xiang B (2025) Longitudinal association between ADL disability and depression in middle-aged and elderly: National cohort study. J Nutr Health Aging 29(2):100450. 10.1016/j.jnha.2024.100450 39674106 10.1016/j.jnha.2024.100450 PMC12180056

[39] Dapp U, Minder CE, Golgert S, Klugmann B, Neumann L, von Renteln-Kruse W (2021) The inter-relationship between depressed mood, functional decline and disability over a 10-year observational period within the longitudinal urban cohort ageing study (LUCAS). J Epidemiol Commun Health 75(5):450–457. 10.1136/jech-2020-214168 10.1136/jech-2020-214168 PMC8053334 33158941

[40] Aidoud A, Gana W, Poitau F, Debacq C, Leroy V, Nkodo JA et al (2023) High prevalence of geriatric conditions among older adults with cardiovascular disease. J Am Heart Assoc 12(2):e026850. 10.1161/JAHA.122.026850 36628962 10.1161/JAHA.122.026850 PMC9939057

[41] Keeney T, Fox AB, Jette DU, Jette A (2019) Functional trajectories of persons with cardiovascular disease in late life. J Am Geriatr Soc 67(1):37–42. 10.1111/jgs.15584 30460975 10.1111/jgs.15584 PMC6705121

[42] Agarwal S, Ozor IN, Chithanuru S, Odumosu EO, Fadiora OE, Ikwan G et al (2025) Optimizing cardiovascular care in aging populations: A comprehensive review of geriatric cardiology. Cureus 17(7):e87992. 10.7759/cureus.87992 40821317 10.7759/cureus.87992 PMC12352464

[43] Salinas-Rodriguez A, Rojas-Botero ML, Rivera-Almaraz A, Fernandez-Nino JA, Montanez-Hernandez JC, Manrique-Espinoza B (2024) Long-term inequalities in health among older Mexican adults: an outcome-wide analysis. SSM Popul Health 26:101684. 10.1016/j.ssmph.2024.101684 38881818 10.1016/j.ssmph.2024.101684 PMC11179325

[44] Lopez-Leon S, Wegman-Ostrosky T, Perelman C, Sepulveda R, Rebolledo PA, Cuapio A et al (2021) More than 50 long-term effects of COVID-19: a systematic review and meta-analysis. Sci Rep 11(1):16144. 10.1038/s41598-021-95565-8 34373540 10.1038/s41598-021-95565-8 PMC8352980

[45] Betschart M, Rezek S, Unger I, Ott N, Beyer S, Boni A et al (2021) One year follow-up of physical performance and quality of life in patients surviving COVID-19: a prospective cohort study. Swiss Med Wkly 151:w30072. 10.4414/smw.2021.w30072 34751538 10.4414/smw.2021.w30072

[46] Jung J, Kim S, Kim B, Kim M, Yang J, Chung D et al (2022) Accelerated cognitive function decline in Community-Dwelling older adults during COVID-19 pandemic: the Korean frailty and aging cohort study (KFACS). Int J Environ Res Public Health 19(17). 10.3390/ijerph191710666 10.3390/ijerph191710666 PMC9518427 36078381

[47] Bello-Chavolla OY, Gonzalez-Diaz A, Antonio-Villa NE, Fermin-Martinez CA, Marquez-Salinas A, Vargas-Vazquez A et al (2021) Unequal impact of structural health determinants and comorbidity on COVID-19 severity and lethality in older Mexican adults: considerations beyond chronological aging. J Gerontol Biol Sci Med Sci 76(3):e52–e9. 10.1093/gerona/glaa163 10.1093/gerona/glaa163 PMC7337730 32598450

[48] Basto-Abreu A, Carnalla M, Torres-Ibarra L, Romero-Martinez M, Martinez-Barnetche J, Lopez-Martinez I et al (2022) Nationally representative SARS-CoV-2 antibody prevalence estimates after the first epidemic wave in Mexico. Nat Commun 13(1):589. 10.1038/s41467-022-28232-9 35105873 10.1038/s41467-022-28232-9 PMC8807586

[49] Oran DP, Topol EJ (2020) Prevalence of asymptomatic SARS-CoV-2 infection: A narrative review. Ann Intern Med 173(5):362–367. 10.7326/M20-3012 32491919 10.7326/M20-3012 PMC7281624

[50] Ayoubkhani D, Khunti K, Nafilyan V, Maddox T, Humberstone B, Diamond I et al (2021) Post-covid syndrome in individuals admitted to hospital with covid-19: retrospective cohort study. BMJ 372:n693. 10.1136/bmj.n693 33789877 10.1136/bmj.n693 PMC8010267

[51] Katsoularis I, Fonseca-Rodriguez O, Farrington P, Jerndal H, Lundevaller EH, Sund M et al (2022) Risks of deep vein thrombosis, pulmonary embolism, and bleeding after covid-19: nationwide self-controlled cases series and matched cohort study. BMJ 377:e069590. 10.1136/bmj-2021-069590 35387772 10.1136/bmj-2021-069590 PMC8984137

[52] Bricio-Barrios JA, Rios-Silva M, Huerta M, Cardenas-Maria RY, Garcia-Ibanez AE, Diaz-Mendoza MG et al (2022) Impact on the nutritional and functional status of older Mexican adults in the absence of recreational activities due to COVID-19: A longitudinal study from 2018 to 2021. J Appl Gerontol 41(9):2096–2104. 10.1177/07334648221099278 35503553 10.1177/07334648221099278 PMC9066228

[53] Cabrero Castro JE, Wong R, Samper Ternent R, Downer B (2024) Population-level trends in self-reported healthcare utilization among older adults in Mexico with and without cognitive impairment. BMC Geriatr 24(1):652. 10.1186/s12877-024-05247-z 39095702 10.1186/s12877-024-05247-z PMC11295330

[54] Bautista-Reyes D, Werner-Sunderland J, Aragón-Gama AC, Duran JRC, Medina KDC, Urbina-Fuentes M et al (2023) Health-care policies during the COVID-19 pandemic in mexico: A continuous case of heterogeneous, reactive, and unequal response. Health Policy OPEN 5:100100. 10.1016/j.hpopen.2023.100100 37662095 10.1016/j.hpopen.2023.100100 PMC10471918

