# Supplementary material for: Impact of the SARS-CoV-2 pandemic on healthy aging and functionality in older Mexican adults: insights from the MHAS cohort
Source: Aging Clin Exp Res. 2026 Feb 4;38(1):81. doi: 10.1007/s40520-026-03333-3 (PMC12901177; doi:10.1007/s40520-026-03333-3)
Supplement: Supplementary file 1 — Supplementary Material 1 [file 40520_2026_3333_MOESM1_ESM.docx]

**Supplementary Material 1. MHAS Variable Operationalization for the Healthy Aging Score (ATHLOS Framework)**

The Healthy Aging Score was constructed following the ATHLOS methodology, using harmonized variables that capture cognitive, sensory, psychological, and functional capacity dimensions. Each item was dichotomized to indicate presence vs. absence of difficulty, and a two-parameter logistic Item Response Theory (IRT) model was applied. Items traditionally included in the ATHLOS framework were mapped to their closest conceptual equivalents in the MHAS/ENASEM 2021 questionnaire.

When an ATHLOS variable did not exist in MHAS, a conceptually comparable variable was identified (e.g., near vision, numeracy, housework). Variables without a valid functional analogue (e.g., dizziness, map use, far-distance hearing in conversation) were not incorporated to avoid contamination. Importantly, **Activities of Daily Living (ADLs), Instrumental Activities of Daily Living (IADLs), and chronic diseases are not part of the HAS** and were analyzed separately.

| **ATHLOS Harmonized Variable** | **MHAS Question** | **Response Options** | **Harmonization Rule (Presence of Difficulty)** |
| --- | --- | --- | --- |
| **Memory** | How would you rate your memory at the present time? | 1 Excellent – 5 Poor | 1–3 Absence; 4–5 Presence |
| **Immediate recall** | Immediate recall list | 0–8 | ≤25th percentile Presence; >25 Absence |
| **Delayed recall** | Delayed recall list | 0–8 | ≤25th percentile Presence; >25 Absence |
| **Verbal fluency** | Animal naming | Continuous | ≤25th percentile Presence |
| **Orientation in time** | Orientation questions | Excellent–Poor | 1–3 Absence; 4–5 Presence |
| **Processing speed** | Visual scanning | 0–60 | ≤25th percentile Presence |
| **Sleeping** | Restless sleep | Yes / No | Yes Presence |
| **Pain** | Pain | Yes / No | Yes Presence |
| **Energy** | Low energy | Yes / No | No energy Presence |
| **Urine incontinence** | Urinary loss | Yes / No | Yes Presence |
| **Eyesight** | Vision (with glasses) | Excellent–Legally blind | 1–3 Absence; 4–6 Presence |
| **Hearing general** | Hearing ability | Excellent–Legally deaf | 1–3 Absence; 4–6 Presence |
| **Stooping/Kneeling** | Difficulty stooping/kneeling | Yes / No / Can’t do | Yes / Can’t do Presence |
| **Lifting/Carrying** | Picking up heavy objects | Yes / No / Can’t do | Yes / Can’t do Presence |
| **Climbing stairs** | Long stairs | Yes / No / Can’t do | Yes / Can’t do Presence |
| **Getting up** | Getting up from sitting | Yes / No / Can’t do | Yes / Can’t do Presence |
| **Walking** | Walking blocks | Yes / No / Can’t do | Yes / Can’t do Presence |
| **Pulling/Pushing** | Pulling large objects | Yes / No / Can’t do | Yes / Can’t do Presence |
| **Sitting long** | Sitting 2 hours | Yes / No / Can’t do | Yes / Can’t do Presence |
| **Reaching arms** | Extending arms | Yes / No / Can’t do | Yes / Can’t do Presence |
| **Walking speed** | 4-meter walk test | Continuous | Slowest quartile Presence |
| **Picking up** | Picking up coin | Yes / No / Can’t do | Yes / Can’t do Presence |
| **Getting in/out of bed** | Difficulty getting in/out of bed | Yes / No / Can’t do | Yes / Can’t do Presence |
| **Bathing** | Difficulty bathing | Yes / No / Can’t do | Yes / Can’t do Presence |
| **Dressing** | Difficulty dressing | Yes / No / Can’t do | Yes / Can’t do Presence |
| **Moving across room** | Walking across room | Yes / No / Can’t do | Yes / Can’t do Presence |
| **Toilet** | Toilet use difficulty | Yes / No / Can’t do | Yes / Can’t do Presence |
| **Eating** | Difficulty eating | Yes / No / Can’t do | Yes / Can’t do Presence |
| **Shopping** | Grocery shopping difficulty | Yes / No / Can’t do | Yes / Can’t do Presence |
| **Preparing meals** | Preparing hot meal | Yes / No / Can’t do | Yes / Can’t do Presence |
| **Managing money** | Managing money | Yes / No / Can’t do | Yes / Can’t do Presence |
| **Taking medications** | Taking medicines | Yes / No / Can’t do | Yes / Can’t do Presence |
