# Supplementary material for: Impact of the SARS-CoV-2 pandemic on healthy aging and functionality in older Mexican adults: insights from the MHAS cohort
Source: Aging Clin Exp Res. 2026 Feb 4;38(1):81. doi: 10.1007/s40520-026-03333-3 (PMC12901177; doi:10.1007/s40520-026-03333-3)
Supplement: Supplementary file 2 — Supplementary Material 2 [file 40520_2026_3333_MOESM2_ESM.docx]

**Supplementary Material 2**

*Regression Coefficients and 95% Confidence Intervals for Healthy Aging Score (HAS) Models*

| **Variable** | | **Original Model (RE)** | **Excluding Key Variables** | **Excluding Participants Aged 80+** | **Excluding Extreme Values** | **Including Interaction Terms for Vaccination** | **Fixed-Effects Model** |
| --- | --- | --- | --- | --- | --- | --- | --- |
| **Female** | | -2.09 (-2.45, -1.73) | -2.01 (-2.40, -1.62) | -2.15 (-2.50, -1.80) | -2.04 (-2.38, -1.70) | -2.00 (-2.35, -1.65) | -2.02 (-2.37, -1.67) |
| **Year** | | -0.34 (-0.51, -0.17) | -0.31 (-0.48, -0.14) | -0.36 (-0.53, -0.19) | -0.32 (-0.49, -0.15) | -0.30 (-0.47, -0.13) | -0.33 (-0.50, -0.16) |
| **Age group** | **70-79** | -0.12 (-0.60, 0.37) | -0.14 (-0.62, 0.34) |  | -0.10 (-0.58, 0.38) | -0.11 (-0.59, 0.37) | -0.13 (-0.61, 0.35) |
|  | **80-89** | -1.44 (-2.34, -0.54) | -1.50 (-2.40, -0.60) |  | -1.38 (-2.28, -0.48) | -1.40 (-2.30, -0.50) | -1.42 (-2.32, -0.52) |
| **Income quartile** | **Q2** | -0.82 (-1.21, -0.44) | -0.80 (-1.18, -0.42) | -0.85 (-1.24, -0.46) | -0.79 (-1.17, -0.41) | -0.81 (-1.19, -0.43) | -0.83 (-1.21, -0.45) |
|  | **Q3** | -1.07 (-1.52, -0.62) | -1.10 (-1.55, -0.65) | -1.12 (-1.57, -0.67) | -1.05 (-1.50, -0.60) | -1.08 (-1.53, -0.63) | -1.09 (-1.54, -0.64) |
|  | **Q4** | -0.50 (-1.06, 0.07) | -0.48 (-1.04, 0.09) | -0.52 (-1.08, 0.05) | -0.47 (-1.03, 0.10) | -0.49 (-1.05, 0.08) | -0.51 (-1.07, 0.06) |
| **Mean age** | | -2.82 (-3.33, -2.31) | -2.80 (-3.31, -2.29) | -2.85 (-3.36, -2.34) | -2.81 (-3.32, -2.30) | -2.83 (-3.34, -2.32) | -2.84 (-3.35, -2.33) |
| **Mean income quartile** | | 1.09 (0.83, 1.36) | 1.08 (0.82, 1.35) | 1.10 (0.84, 1.37) | 1.07 (0.81, 1.34) | 1.06 (0.80, 1.33) | 1.09 (0.83, 1.36) |
| **Depression** | | -5.17 (-5.47, -4.86) |  | -5.10 (-5.40, -4.80) | -5.12 (-5.42, -4.82) | -5.15 (-5.45, -4.85) | -5.18 (-5.48, -4.88) |
| **Hypertension** | | -1.58 (-1.88, -1.28) |  | -1.55 (-1.85, -1.25) | -1.60 (-1.90, -1.30) | -1.57 (-1.87, -1.27) | -1.59 (-1.89, -1.29) |
| **Diabetes** | | -2.03 (-2.37, -1.69) |  | -2.00 (-2.34, -1.66) | -2.05 (-2.39, -1.71) | -2.02 (-2.36, -1.68) | -2.04 (-2.38, -1.70) |
| **Coronary heart disease** | | -2.71 (-3.18, -2.24) | -2.68 (-3.15, -2.21) | -2.75 (-3.22, -2.28) | -2.69 (-3.16, -2.22) | -2.70 (-3.17, -2.23) | -2.72 (-3.19, -2.25) |
| **Obesity** | | -1.09 (-1.48, -0.71) | -1.08 (-1.47, -0.70) | -1.10 (-1.49, -0.72) | -1.07 (-1.46, -0.69) | -1.06 (-1.45, -0.68) | -1.09 (-1.48, -0.71) |
| **Stroke** | | -3.13 (-4.03, -2.22) | -3.10 (-4.00, -2.19) | -3.15 (-4.05, -2.24) | -3.11 (-4.01, -2.20) | -3.12 (-4.02, -2.21) | -3.14 (-4.04, -2.23) |
| **Exercise** | | 1.46 (1.18, 1.74) | 1.50 (1.22, 1.78) | 1.42 (1.14, 1.70) | 1.48 (1.20, 1.76) | 1.44 (1.16, 1.72) | 1.45 (1.17, 1.73) |
| **Employment status** | | 1.52 (1.20, 1.84) | 1.55 (1.23, 1.87) | 1.50 (1.18, 1.82) | 1.53 (1.21, 1.85) | 1.51 (1.19, 1.83) | 1.54 (1.22, 1.86) |
| **COVID** | | -0.25 (-0.78, 0.28) | -0.26 (-0.79, 0.27) | -0.27 (-0.80, 0.26) | -0.24 (-0.77, 0.29) | -0.23 (-0.76, 0.30) | -0.26 (-0.79, 0.27) |
| **COVID hospitalization** | | -1.96 (-3.65, -0.26) | -1.98 (-3.67, -0.28) | -1.95 (-3.64, -0.25) | -1.97 (-3.66, -0.27) | -1.94 (-3.63, -0.24) | -1.96 (-3.65, -0.26) |
| **COVID vaccination** | | 0.49 (-0.03, 1.01) | 0.48 (-0.04, 1.00) | 0.50 (-0.02, 1.02) | 0.47 (-0.05, 0.99) | 0.46 (-0.06, 0.98) | 0.48 (-0.04, 1.00) |

**Supplementary Material 2: Odds Ratios and 95% Confidence Intervals for Functional Decline Models**

| **Variable** | | **Original Model (RE)** | **Excluding Key Variables** | **Excluding Participants Aged 80+** | **Excluding Extreme Values** | **Including Interaction Terms for Vaccination** | **Fixed-Effects Model** |
| --- | --- | --- | --- | --- | --- | --- | --- |
| **Female** | | 1.24 (1.07, 1.45) | 1.21 (1.05, 1.42) | 1.26 (1.09, 1.48) | 1.23 (1.06, 1.44) | 1.22 (1.05, 1.43) | 1.23 (1.06, 1.44) |
| **Year** | | 1.22 (1.13, 1.32) | 1.21 (1.12, 1.31) | 1.23 (1.14, 1.33) | 1.20 (1.11, 1.30) | 1.21 (1.12, 1.31) | 1.22 (1.13, 1.32) |
| **Age group** | **70-79** | 0.89 (0.68, 1.16) | 0.87 (0.67, 1.14) |  | 0.86 (0.66, 1.13) | 0.88 (0.68, 1.15) | 0.89 (0.69, 1.16) |
|  | **80-89** | 1.27 (0.89, 1.81) | 1.30 (0.92, 1.85) |  | 1.25 (0.87, 1.78) | 1.26 (0.88, 1.80) | 1.28 (0.90, 1.83) |
| **Income quartile** | **Q2** | 1.22 (1.01, 1.47) | 1.21 (1.00, 1.46) | 1.23 (1.02, 1.48) | 1.20 (0.99, 1.45) | 1.21 (1.00, 1.46) | 1.22 (1.01, 1.47) |
|  | **Q3** | 1.27 (1.02, 1.58) | 1.29 (1.04, 1.60) | 1.31 (1.06, 1.62) | 1.26 (1.01, 1.57) | 1.27 (1.02, 1.58) | 1.28 (1.03, 1.59) |
|  | **Q4** | 1.06 (0.81, 1.40) | 1.05 (0.80, 1.38) | 1.07 (0.82, 1.41) | 1.04 (0.79, 1.37) | 1.05 (0.80, 1.38) | 1.06 (0.81, 1.39) |
| **Mean age** | | 1.19 (1.05, 1.37) | 1.18 (1.04, 1.36) | 1.20 (1.06, 1.38) | 1.17 (1.03, 1.35) | 1.18 (1.04, 1.36) | 1.19 (1.05, 1.37) |
| **Mean income quartile** | | 0.88 (0.72, 1.04) | 0.87 (0.71, 1.03) | 0.89 (0.73, 1.05) | 0.86 (0.70, 1.02) | 0.87 (0.71, 1.03) | 0.88 (0.72, 1.04) |
| **Depression** | | 3.89 (3.14, 4.72) |  | 3.87 (3.12, 4.70) | 3.85 (3.10, 4.68) | 3.88 (3.13, 4.71) | 3.90 (3.15, 4.73) |
| **Hypertension** | | 1.34 (1.10, 1.63) |  | 1.33 (1.09, 1.62) | 1.35 (1.11, 1.64) | 1.34 (1.10, 1.63) | 1.33 (1.09, 1.62) |
| **Diabetes** | | 1.83 (1.52, 2.11) |  | 1.80 (1.50, 2.09) | 1.82 (1.51, 2.10) | 1.83 (1.52, 2.11) | 1.84 (1.53, 2.12) |
| **Coronary heart disease** | | 1.96 (1.52, 2.54) | 1.95 (1.51, 2.52) | 1.98 (1.54, 2.57) | 1.94 (1.50, 2.50) | 1.95 (1.51, 2.52) | 1.96 (1.52, 2.53) |
| **Obesity** | | 1.39 (1.14, 1.70) | 1.38 (1.13, 1.69) | 1.40 (1.15, 1.71) | 1.37 (1.12, 1.68) | 1.38 (1.13, 1.69) | 1.39 (1.14, 1.70) |
| **Stroke** | | 2.64 (1.93, 3.58) | 2.63 (1.92, 3.57) | 2.65 (1.94, 3.60) | 2.62 (1.91, 3.55) | 2.63 (1.92, 3.57) | 2.64 (1.93, 3.58) |
| **Exercise** | | 0.60 (0.46, 0.75) | 0.59 (0.45, 0.74) | 0.61 (0.47, 0.76) | 0.58 (0.44, 0.73) | 0.59 (0.45, 0.74) | 0.60 (0.46, 0.75) |
| **Employment status** | | 0.52 (0.39, 0.65) | 0.53 (0.40, 0.66) | 0.54 (0.41, 0.67) | 0.51 (0.38, 0.64) | 0.52 (0.39, 0.65) | 0.53 (0.40, 0.66) |
| **COVID** | | 0.86 (0.66, 1.12) | 0.85 (0.65, 1.11) | 0.87 (0.67, 1.13) | 0.84 (0.64, 1.10) | 0.85 (0.65, 1.11) | 0.86 (0.66, 1.12) |
| **COVID hospitalization** | | 1.36 (0.89, 2.08) | 1.35 (0.88, 2.06) | 1.37 (0.90, 2.10) | 1.34 (0.87, 2.04) | 1.35 (0.88, 2.06) | 1.36 (0.89, 2.08) |
| **COVID vaccination** | | 0.75 (0.56, 0.92) | 0.74 (0.55, 0.91) | 0.76 (0.57, 0.93) | 0.73 (0.54, 0.90) | 0.74 (0.55, 0.91) | 0.75 (0.56, 0.92) |
